# Items

**Published:** 1883-12

**Authors:** 


### Items.

"THE LIVING DEATH."—[*The Southern Clinic*, C. A. Bryce, Editor, M.D., Richmond, Va., Oct., 1883. Editorial.]

We received a pamphlet published by Dr. H. H. Kane, of 46 West Fourteenth Street, New York city, and issued to the people of this broad land, far and wide, under the above caption.

On account of the betrayal of professional confidence reposed in him, and on account of the open violation of the Code of Ethics by this Dr. Kane, it becomes our duty to denounce this man as unworthy of recognition by the profession. A brief *résumé* of the contents of *The Living Death* will serve to establish the grounds of our denunciation.

In the "introduction," Dr. Kane announces his great experience in the treatment of opium, chloral, morphia, hashisch, and alcohol habitués, and claims to have at last *discovered* a combination by which any one may be speedily, painlessly, and secretly cured at his or her home by using his preparations ; the formula and ingredients are, however, kept secret. He also claims to be so very careful of the communications of all applying to him for medicine, that he has adopted the plan of destroying all letters sent to him. He, after mature deliberation and upon the urgent solicitation of members of the profession all over the country, has consented to issue this circular broadcast to the people to let them know where this inestimable boon may be procured, and finally in order to convince those who are unacquainted with his name and high professional attainments, as to the responsibility of his statements, he has introduced letters and testimonials from the most eminent men of America and England.

This "little brochure" is indeed "the crowning effort" of an unprofessional career. Dr. Kane has already, just within the pale of the code, advertised himself by his publications, and in the last year he has utilized the daily papers of New York and the illustrated periodicals to extend his fame as a specialist. To-day he goes beyond all previous efforts by announcing himself the sole proprietor of a secret remedy; this is bad enough, but he is also bold enough to use in support of his treatment professional endorsements, selected from private correspondence in the past, when this Dr. Kane was a legitimate physician practicing a specialty. As we read these commendatory letters we are surprised at the insolence of the man who dares use his eminent correspondents to advance his advertising scheme, and we find nowhere in these letters any approval of his secret treatment, or any recognition of his ownership of a specific.

From the "drugs that enslave" to *The Living Death* is a long step, the step from scientific labor to charlatanism, and Dr. Kane cannot honestly use letters written to the author of his books to advertise the proprietor of a secret remedy for the habits which he terms a Living Death.

He has voluntarily placed himself among the "cancer doctors," patent medicine men, and quacksalvers generally who flood the country with just such literature as this *Living Death*, and in advertising his nostrum he avows himself no longer worthy professional countenance or support, no longer a scientific physician.

Every paragraph of this remarkable production seems to have been modeled after the blood-curdling style of the "Private Diseases Cured" quack, who first scares his patient into the disease he professes to cure and then makes a fee out of him. To this ignoble company, as to the communion of choice spirits, we commend Dr. H. H. Kane.

We will briefly allude to a particulary unethical proceeding in this advertising pamphlet, we mean the attack on Dr. J. B. Mattison, of Brooklyn, and the use of a private letter from Dr. Mattison to contrast the comparative ability of Mattison and Kane. In this, Kane's charlatanism contrasts most unfavorably with the candor of a gentleman who does not own a secret nostrum, but who has a wide reputation for intelligence, ability and as an honorable and ethical practitioner.

---

OFFICIAL LIST OF CHANGES OF STATIONS AND DUTIES OF MEDICAL OFFICERS OF THE UNITED STATES MARINE HOSPITAL SERVICE. JULY 1, 1883 TO SEPTEMBER 30, 1883.

Bailhache, P. H., Surgeon: detailed as member of Board to examine candidates for promotion, Aug. 23, 1883; detailed as Surgeon-in-charge Cape Charles Quarantine Station, Sept. 5, 1883.

Miller, T. W., Surgeon: granted leave of absence for twenty-five days, August 31, 1883.

Wyman, Walter, Surgeon: detailed as member of Board to examine candidates for promotion, August 23, 1883.

Long, W. H., Surgeon: granted leave of absence for twenty days, Sept. 25, 1883.

Smith, Henry, Surgeon: directed to take charge of quarantine service at the Capes, July 29, 1883.

Stoner, G. W., Passed Assistant Surgeon: granted leave of absence for thirty days, Aug. 24, 1883; to inspect the relief stations along the coast of Maine, Sept. 29, 1883.

Goldsborough, C. B., Passed Assistant Surgeon: granted leave of absence for thirty days, Aug. 29, 1883.

Banks, C. E., Assistant Surgeon: relieved from duty at Portland, Oregon, and to report to the Surgeon-General at Washington, July 10, 1883.

Carmichael, D. A., Assistant Surgeon: granted leave of absence for ten days, Aug. 31, 1883.

Peckham, C. T., Assistant Surgeon: to proceed to Portland, Maine, for temporary duty, Aug. 25, 1883.

Devan, S. C., Assistant Surgeon: to proceed to Portland, Oregon and assume charge of the Service, Sept. 11, 1883.

Kallock, P. C., Assistant Surgeon: to proceed to Philadelphia, Pa., for temporary duty, July 25, 1883; to rejoin his station (New York) July 31, 1883.

Yemans, H. W., Assistant Surgeon: relieved from duty at Sitka,

Alaska, and to proceed to Portland, Oregon, for temporary duty, July 10, 1883; to proceed to San Francisco, Cal., reporting for duty to Surgeon Vansant, Sept. 11, 1883.

Glennan, A. H., Assistant Surgeon: to remain at Norfolk, Va., until further orders, July 29, 1883.

Wasdin, Eugene, Assistant Surgeon: to proceed to New Orleans, La., for temporary duty, Aug. 2, 1883; to proceed to Mobile, Ala., for temporary duty, Aug. 27, 1883; to rejoin his station (New Orleans) as soon as practicable, September 25, 1883.

PROMOTIONS.

Guitèras, John, Passed Assistant Surgeon: promoted and appointed Passed Assistant Surgeon by the Secretary of the Treasury from September 1, 1883. August 31, 1883.

Wheeler, W. A., Passed Assistant Surgeon: promoted and appointed Past Assistant Surgeon by the Secretary of the Treasury from September 1, 1883. August 31, 1883.

RESIGNATION.

O'Connor, F. J., Assistant Surgeon: resignation accepted by the Secretary of the Treasury, to take effect August 1, 1883. August 2, 1883.

APPOINTMENT.

Wasdin, Eugene, M.D., of South Carolina, having passed the examination required by the regulations, was appointed an assistant Surgeon by the Secretary of the Treasury, August 2, 1883.

---

A DOCTOR'S SANCTUM.

In an article entitled "Rab's Friend," published in the *Century Magazine*, the following description is given of the study of the late Dr. John Brown, of Edinburgh, the well-known author of "Rab and His Friends:"

"The room is crowded with pictures, engravings, books and pretty things of all kinds—nothing, perhaps, very valuable—but nearly all of them with a history; nearly all given to him in one way or another by some one who admired or loved him. The frame in the middle line on the extreme right of the fireside contains five of Leech's little original drawings; and there is a famous larger one on the same line beyond the head of Tennyson, while beneath is the grand Goldsmith "in his new suit," from Thackeray's "Humorists." The black picture diagonally to the right above him contains the original sketch, by George Harvey, of the girl's head (including his daughter and Dr. Brown's,) made for his picture of "Leaving the Manse," and afterward utilized as "Rab's Friends" in the illustrated edition. Above the bust to the left is the best of the heads of "Rab" by the very clever young animal painter, Edwin Douglass. The white frame to the left of the gas globe shows, by a slight penciled profile likeness, the beautiful head of Mrs. Brown in her prime; that, partially concealed by the other gas globe, is her likeness as a young girl, and immediately below it is her bust; the other bust on the mantelpiece being that of Dr. John Scott, the Edinburgh physician, while the portrait behind is that of John Scott, of the *London Magazine*, who was an uncle or cousin of Mrs. Brown. The engraving in the middle line, immediately to the left of the mantelpiece, is Turner's "Devil's Bridge," over the Reuss, in the Altdorf valley, and the bull-dog's head to the left of it (a fine engraving) bears the inscription, "Not an approach to the great Rab, but—yours, truly, E. Landseer." The picture to the left of young John's bust are, first, a very beautiful and characteristic landscape of Harvey's, much akin in character to that engraved in the illustrated Rab for "Rab's Grave;" and, next, a small but very good likeness of old Dr. Brown, Dr. Brown's father. There is also a wonderfully clever spaniel dog drawn by Turner, and a drawing by Ruskin, both being, I think, presented by Ruskin. Of the books on the table may be noticed two volumes of O. W. Holmes' poetical works, the gift of the "Autocrat," with a letter which gave Dr. Brown as much pleasure as anything which had come to him for years."

---

THE CHICAGO THROAT AND CHEST HOSPITAL.

A meeting was held recently at the residence of the Rev. William Lawrence for the purpose of organizing in Chicago a

Throat and Chest Hospital. Quite a number of prominent gentlemen were present, and matters were so far advanced that a Board of Directors was chosen, being made up of the following gentlemen: The Hon. Wm. Aldrich, the Rev. Wm. Lawrence, Dr. E. Fletcher Ingals, Dr. G. F. Hawley, and Messrs. C. C. Kohlsaat, W. Howard and Herman Kohlsaat. The hospital will be conducted after the manner of similar institutions in Europe. Persons who are able pay for the service of a physician will not be admitted, the hospital being designed for the accommodation of patients who can only afford small weekly payments, according to their means. The fees and donations will go to the hospital fund. There will be an outdoor department for the treatment of patients, ard everything is expected to be ready in a very few weeks. We have made arrangements for the care of in-patients who are only able to pay for board. The outdoor department will be in running order about December 1st.

---

WE regret very much that the account of the last illness of the talented young Dr. Rea, written by his uncle, Prof. Rea, was not published in the last JOURNAL. The article was lost in the midst of a change that took place in the disposition of forces in the printer's office. It has since been found and appears in this number.

---

PRESENTATION OF THE CHILD'S HEAD BY THE VULVA AND ANUS.

A primapara, 28 years of age, was attended by a midwife, and the labor pains were regular. When the head progressed, the perinæum was found to be resistant, and the pains continued for 12 hours without effect. Then a physician was sent for, who found the recto-vaginal tissues entirely destroyed, and the head was visible through the dilated anus, as well as through the vulva. The applied forceps soon delivered the child. It is advisable in those cases to apply the forceps without delay.—*Gazette Médicale de Nantes.*

